# Leadership and well-being of employees in the Nordic countries: A literature review

**DOI:** 10.3233/WOR-210063

**Published:** 2023-04-18

**Authors:** Daniel Lundqvist, Andreas Wallo, Cathrine Reineholm

**Affiliations:** a Department of Behavioural Sciences and Learning, Linköping University, Linköping, Sweden; b HELIX Competence Centre, Linköping University, Linköping, Sweden

**Keywords:** Manager, supervisor, employee, health, Nordic countries

## Abstract

**BACKGROUND:**

There is a need for more knowledge regarding the importance of managerial leadership for fostering well-being in the workplace and how context has been accounted for in previous research.

**OBJECTIVE:**

To carry out a literature review of previous research that empirically examines the importance of leadership for well-being in a Nordic working life context.

**METHODS:**

A rapid literature review was conducted with narrative analysis in 5 steps: establish focus, research questions, and inclusion criteria; literature search; relevance screening; quality assessment; data analysis. The search identified 4566 unique studies where 35 quantitative and five qualitative met the relevance and quality criteria.

**RESULTS:**

Findings from quantitative and qualitative studies are presented. Transformational and supportive leadership are recurrently associated with employee well-being, although the qualitative studies also highlight adaptive leadership and leaders being available and providing space. Some connections are made to the Nordic context in the reviewed studies, but these connections are not fully elaborated.

**CONCLUSION:**

Leadership is related to employee well-being, although this relationship seems to be indirect, mediated by other factors in the working environment. The review identifies the need for more well-designed studies addressing the contextual factors of this relationship, and how leadership should be exercised in practice.

## 1 Introduction

Traditionally, the importance of leadership in organisations has been studied in relation to performance measurement indicators such as earnings, efficiency, productivity, and quality [1, 2]. In recent years the interest in studies of how managerial leadership influences employee well-being in the workplace has grown considerably [3]. Previous research has investigated how managers indirectly coordinate and encourage different kinds of activities that promote well-being (e.g., participation in wellness activities) [4], and how managers can create work environments that are conducive to employee well-being, for example, through how the work is organised [4, 5]. However, studies of how leaders can promote well-being among employees through their *direct behaviour and leadership style* constitute a relatively new research field and the clearer connection to well-being or the framing of a health perspective has not yet been fully explored in empirical studies [4, 6, 7].

Previous literature reviews and meta-analyses [8–14] of the existing empirical findings have concluded that there is an association between leadership styles and various measures of well-being. For example, these studies show that transformational leadership [8–11, 13], high rates of task- and relationship-oriented leadership [8–10, 12], and high-quality interactions between leaders and employees are associated with employees’ well-being [10–12], either directly or indirectly through other factors. In addition, the most recent literature review by Inceoglu et al. [14] explored positive leadership behaviour and leadership styles relative to employee well-being. However, in their review, they only included empirical studies investigating mediators. The review demonstrated that it is often positive forms of well-being (e.g. job satisfaction) that have been studied, with a particular focus on mental or emotional well-being, and less of a focus on physical well-being. The review further found that primarily neo-charismatic leadership, such as transformational leadership [15], had been studied in relation to employee well-being, such as job satisfaction. The relationship between leadership and well-being was mediated by social cognitive (e.g. perceived competence) or relational mediators (e.g. social support). Interestingly, none of the critiques of transformational leadership from prominent scholars in the field [16–18] was brought up in the review. We argue it is important to not only examine the potential associations between leadership and well-being, but also the theoretical foundation of the research in relation to the context in which it was conducted.

When taken together, the previous attempts at reviewing the state of art in the field points to several problems with the existing empirical research [8–14]. Much of the research is still cross-sectional making it impossible to distinguish the direction of the relationship, and more high-quality longitudinal studies, with data from several different sources, is called for. Moreover, there has been little attention given to understanding the process of how leadership affects well-being, and on the role of context in the relationship between leadership and well-being. The focus in previous research has often been on establishing a relationship between leadership and well-being, and there is a need for more knowledge regarding how context influence this relationship [3]. The need for more qualitative studies and the use of standardised instruments has also been emphasised [10]. A better understanding of why, for whom, and when leadership is important is needed, and not merely how strong this relationship is[3, 10].

Despite this common concern for more knowledge about the influence of context, it is striking that previous literature reviews have not included qualitative studies that may capture contextual aspects more easily, even though this has been suggested for several years [10]. Furthermore, previous reviews have not clarified how the empirical studies have related to the fact that the studies were conducted in certain environments and with certain participants. Previous reviews have hardly paid attention to the samples of the reviewed studies, i.e., who has participated in the research, from which organisations, professions, and countries.

In this paper, we address the above critique of previous literature reviews by conducting a review of empirical findings related to a specific context – a Nordic working life context. Our intent is to examine how this context have been accounted for in previous research, and draw attention to how the Nordic context may increase our understanding of the relationship between leadership and well-being. This specific context was chosen for several reasons. First, the context is fairly delineated because the Nordic countries share a common cultural-historical background, with similar social values and a labour market characterised by long-term, consensus-based relationships and inter-corporate networks between its various actors [19–21]. Moreover, the Nordic working life is characterized by the presence of relatively powerful labour unions, a high union density, and the resolution of conflicts in the form of collective agreements between unions and employers. Second, leadership in Nordic countries has been highlighted as being different, emphasising participation, collaboration and self-governance to a higher extent than other cultural contexts [22–24]. In the Swedish leadership model Developmental Leadership [25], which is heavily influenced by Bass’s notion of transformational leadership [26], the concept of charisma has been replaced by inspiration because it appears to induce negative ideas of the leader being superior in the Scandinavian leadership culture [27]. Third, in the Nordic countries, there are also similar, and rather strong, legislation regarding the work environment which requires managers in organisations to work with systematic occupational health and safety reviews [28–30]. Fourth, the previous reviews have shown an availability of empirical studies of leadership and well-being stemming from the Nordic countries. The Nordic countries thus seem to be a sufficiently delimited region and where research relevant to this review has been carried out.

The purpose of this paper is to carry out a literature review of previous research that empirically examines the importance of leadership for well-being in a Nordic working life context.

The following research questions guided our review:

1. Which leadership styles and behaviours have been demonstrated in previous research to be significantly associated with the well-being of employees in a Nordic work-life context?
2. Which theoretical starting points regarding leadership and methods have been used in this research, and in which populations have the research been carried out?
3. How can the theories used, and populations investigated in previous research, be problematized from the context of Nordic working life?

## 2 Method

To answer the research questions, a rapid review was conducted according to Grant and Booth’s classification [31]. The structuring of the review of the papers was staged [32] and guided by the steps for a systematic review proposed in Prisma [33, 34].

First, the content, focus and limitations of the review were established in accordance with the study purpose and research questions. Next, criteria were formulated for which studies to include during the search and review processes. The inclusion criteria were: a) the studies should focus on working life and workplace contexts; b) they should be carried out in a Nordic context; c) they should explore leadership in terms of styles, behaviours, roles and similar concepts or synonyms; d) and they should focus on the relationship between leadership and employee well-being in the workplace (health factors). The studies were also required to be e) scientific articles in international, peer-reviewed (academic) journals; f) written in English; and g) containing empirical material. Studies that did not meet the criteria for inclusion were excluded.

**Fig. 1:**
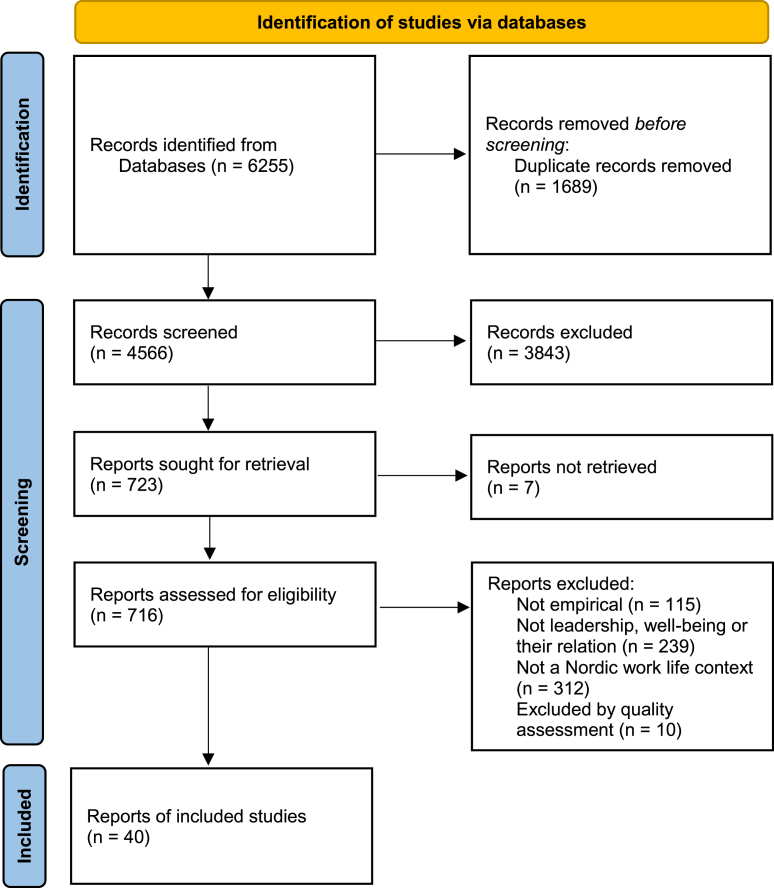
Flowchart of the identification process based on PRISMA.

The searches were conducted in Scopus and Web of Science and produced 4566 unique studies (Fig. 1). Searches were also conducted in Emerald and Business Source but this search yielded no additional studies. Search terms used to capture leadership and well-being were: leadership or leader* behavior* or leader* style* or leader* skills or supervisor* behavior* or LMX or manager* behavior* AND well being or wellbeing or work* health or employ health* or occupational health or subordinate health or healthy employee* or healthy work*. All searches were conducted in August 2020.

The studies were screened based on title and abstract, after which 716 were selected for a review of relevance based on the inclusion criteria for the literature review. Studies that included both positive and negative (salutogenic and pathogenic) indicators of well-being were included, but the focus of this literature review is only on the positive outcomes. For example, a study might investigate leadership in relation to job satisfaction and perceived stress, but the review only includes the results of the relationship between leadership and job satisfaction. The relationship between leadership and well-being was not the main focus of some of the qualitative studies, but studies with findings that at least partly address this relationship were considered relevant. As health and well-being are complex terms that may be defined differently, it was decided to include studies as long as they were not apparently pathogenic, and as long as the authors of the paper themselves considered the outcome to be well-being or health. After reviewing the downloaded full texts for relevance, 50 studies met the inclusion criteria.

These 50 studies underwent a quality assessment based on recognised protocols for quantitative [35, 36] and qualitative studies [37]. Each study was reviewed and graded on a set of quality criteria, resulting in a three-point quality assessment: low, medium-high, or high quality. The quantitative protocol consisted of ten questions (e.g. Was the statistical methodology appropriate for the research question and study design? Are the results interpreted correctly?). The qualitative protocol consisted of five headings with accompanying questions: purpose, selection, data collection, analysis and results. For example, under the heading purpose, one question was: Is the study based on a well-defined problem/question? Of a total of 43 quantitative studies reviewed for quality, 35 were assessed as high or medium-high quality. A common cause for exclusion due to low quality was insufficient statistical processing (e.g. potential confounders were not considered). Of a total of seven qualitative studies reviewed for quality, five were assessed as high or medium-high quality. Thus, a total of 40 studies were included in the review.

The quantitative studies assessed as high or medium-high quality underwent analysis. In the analysis process, all studies were read, and tables were compiled with key information relevant for this literature review. These descriptions can be found in the results section of the literature review, and conclusions are drawn based on these descriptions. This procedure is called narrative synthesis [37].

The qualitative articles were analysed in sequential order, one step at a time. After an initial reading, basic information about the paper, such as journal, country, purpose, etc., was collected. Thereafter, an inductive conventional content analysis [38] of the findings of the papers was carried out. The results, discussion and conclusions were carefully read and, based on this reading, preliminary categories were created from each article. In this step, each article was summarised with a focus on the content and validity of the findings. In the last step, the authors discussed the relationships between the preliminary categories, leading to the identification of overarching categories that were addressed to some degree in most of the included articles.

Each step of the literature review was performed by at least two authors to increase reliability.

## 3 Results

The papers were published between 2008 and 2020, and most were published in the last four years (25 papers). Papers with a quantitative approach will be presented first, followed by those with a qualitative approach. The section for the quantitative papers is structured based on the leadership perspective used in the studies. The section for the qualitative studies starts with a summary of each study and then presents overall patterns found.

### 3.1 Quantitative studies

First, the section describes which leadership theories are associated with which outcome of well-being (see Table 1), followed by an overarching summary of what the quantitative studies demonstrate overall. Studies investigating multiple leadership theories are presented under multiple headings.

**Table 1:** Quantitative studies and health-related outcomes

Paper	Country	Design	Population	Leadership	Health	Bivariate association	Association in final model	Mediators/moderators
Berthelsen et al., 2018	Sweden	Cross-sectional	1,345 employees in dental care in four regions. 90% women.	Supportive	Work ability	Yes	Unclear	Interpersonal resources Task resources Job satisfaction
				Supportive	Job satisfaction	Yes	Yes	Interpersonal resources Task resources
Burr et al., 2010	Denmark	Longitudinal	3,552 randomly selected employees from the population. Gender distribution not specified.	Supportive	Mental health	Not reported	No	Not studied
				Supportive	Vitality	Not reported	No	Not studied
Clausen & Borg, 2011	Denmark	Longitudinal	6,299 employees in public elderly care in 35 municipalities. 96% women.	Supportive	Meaningful work	Yes	Yes	Not studied
Finne et al., 2016	Norway	Longitudinal	4,158 employees in 63 organisations. 60% women.	Supportive	Well-being	Yes	Yes	Not studied
Hagqvist et al., 2018	Sweden	Cross-sectional	379 employees in municipalities and county councils. 82% women.	Supportive	Health	Yes	No	Not studied
				Supportive	Well-being	Yes	No	Not studied
Hasson et al., 2019	Sweden	Cross-sectional	76 managers and 211 employees in forest industry. 18% women.	Transformational	Health	No	No	Not studied
				Transformational	Work ability	No	No	Not studied
				Contingent reward	Health	Yes	No	Not studied
				Contingent reward	Work ability	No	No	Not studied
				Management by exception – passive	Health	No	No	Not studied
				Management by exception – passive	Work ability	Yes	Yes	Not studied
				Laissez-faire	Health	No	No	Not studied
				Laissez-faire	Work ability	No	No	Not studied
Holten et al., 2018	Denmark	Longitudinal	2,947 employees in 35 municipalities. 92% women.	Transformational	Well-being	Yes	Yes (for one subgroup)	Moderator: Ethnicity
					Health	Yes	No	Moderator: Ethnicity
					Job satisfaction	Yes	Yes (for one subgroup)	Moderator: Ethnicity
Kizuki & Fujiwara, 2020	EU	Cross-sectional	28,900 employees in 35 countries. 47% women.	Supportive	Well-being	Not reported	Yes	Social capital
Ljungblad et al., 2014	Sweden	Longitudinal	Employees in 60 randomly selected municipalities. 93% women.	Supportive	Health	Yes (in 1 of 4 items)	No	Social climate Health-promoting activities
Lohela et al., 2009	Sweden	Longitudinal	1,212 employees in four organisations. 14% women.	Supportive	Quality of life	Not reported	Yes	Not studied
Lundmark et al., 2017	Sweden	Cross-sectional	180 white-collar workers in an organisation. 59% women.	Transformational	Health	Yes	No	Intervention leadership
				Transformational	Work ability	No	No	Intervention leadership
				Intervention	Health	Yes	Yes	Unclear
				Intervention	Work ability	Yes	Yes	Unclear
Lundmark et al., 2018	Sweden	Cross-sectional	90 employees in an industrial organisation. 24% women.	Intervention	Work engagement	Yes	No	No mediation
Mauno et al., 2016	Finland	Cross-sectional	3,466 nurses. 89% women.	Transformational	Work engagement	Yes	Yes	Not studied
Munir et al., 2012	Denmark	Longitudinal	188 employees in public elderly care. 93% women.	Transformational	Well-being	Yes	No	Work-life conflicts
				Transformational	Job satisfaction	Yes	Yes	Not mediated
Nie & Lämsä, 2018	Finland	Cross-sectional	117 employees. 41% women.	Benevolent	Job satisfaction	Yes	Yes	Not studied
Nielsen & Daniels, 2012	Denmark	Cross-sectional	425 employees in public elderly care and a private accounting firm. 72% women.	Transformational	Well-being	Yes	Yes	Meaningful work Role conflicts
				Transformational	Job satisfaction	Yes	No	Meaningful work Social support Cohesion Role conflicts
Nielsen, Daniels, Nayani et al., 2019	DK/UK	Cross-sectional	734 employees in 11 organisations. 4% women.	Health and safety leadership	Health	Yes	Yes	Moderator: Lack of isolation
Nielsen & Munir, 2009	Denmark	Longitudinal	188 employees in public elderly care. 93% women.	Transformational	Well-being	Yes	Yes (cross-sectionally)	Self-efficacy (at one time point)
Nielsen & Randall, 2009	Denmark	Cross-sectional	274 employees in public elderly care. 91% women.	Transformational	Well-being	Yes	No	Self-efficacy Team efficacy
				Transformational	Job satisfaction	Yes	Yes	Team efficacy
Nielsen & Randall, 2009	Denmark	Longitudinal	188 employees in elderly care in two organisations. 93% women.	Intervention	Well-being	Yes	No	Work conditions (meaningful work, role clarity, social support)
					Job satisfaction	Yes	No	Work conditions (meaningful work, role clarity, social support)
Nielsen, Randall et al., 2008	Denmark	Longitudinal	188 employees in public elderly care. 93% women.	Transformational	Well-being	Yes	Yes (cross-sectionally)	Role clarity Meaningful work Opportunities for development
Nielsen et al., 2013	Norway	Cross-sectional	464 seafarers. 1% women.	Authentic	Job satisfaction	Yes	Yes (for one subgroup)	Moderator: Ethnicity
Nielsen, Yarker et al., 2008	Denmark	Cross-sectional	447 employees in public elderly care. 93% women.	Transformational	Well-being	Yes	Yes	Involvement Influence Meaningfulness
				Transformational	Job satisfaction	Yes	No	Involvement Influence Meaningfulness
Perko et al., 2016	Finland	Longitudinal	262 employees in the public sector. 88% women.	Transformational	Well-being	Yes	Yes	Not studied
				Authentic	Well-being	Yes	Yes	Not studied
Roczniewska et al., 2020	Sweden	Longitudinal	42 health care units. 89% women.	Transformational	Health	No	No	Not studied
				Transformational	Job satisfaction	Yes	No	Not studied
Svensson et al., 2018	Sweden	Cross-sectional	502 employees at an agency. 39% women.	Task/relations	SOC	Not reported	No	Not studied
Tafvelin, Hasson et al., 2019	Sweden	Longitudinal	211 employees in forestry. 18% women.	Transformational	Job satisfaction	Not reported	Yes	Not studied
Tafvelin et al., 2011	Sweden	Longitudinal	158 randomly selected employees in municipal social services. 79% women.	Transformational	Well-being	Yes	No	Innovation climate
Tafvelin, von Thiele Schwarz, Nielsen et al., 2019	Sweden	Longitudinal	159 hospital employees. 94% women.	Intervention	Work ability	Yes	No	Participation
				Intervention	Job satisfaction	Yes	No	No mediation
Upadyaya & Salmela-Aro, 2020	Finland	Longitudinal	766 employees in three organisations. 48% women.	Servant	Work engagement	Yes	Yes	Not studied
Upadyaya et al., 2016	Finland	Cross-sectional (longitudinal)	1,415 employees in three organisations. 59% women.	Servant	Work engagement	Yes	Yes	Not studied
					Life satisfaction	Yes	Yes	Not studied
van Dick et al., 2018	EU	Cross-sectional	5,290 employees in 20 countries. 53% women.	Transformational	Job satisfaction	Yes	Yes	Not studied
				Authentic	Job satisfaction	Yes	Yes	Not studied
				LMX	Job satisfaction	Yes	Yes	Not studied
				Identity	Job satisfaction	Yes	Yes	Not studied
Westerlund et al., 2010	Sweden	Cross-sectional	12,622 employees in a forestry company (of which 10,384 were in Sweden and Finland). Approx. 14% women among union contract employees, 40–50% women among white-collar employees.	Attentive	Health	Yes	Yes	Not studied
						**Difference t1-t2**	**Difference control group**
Hansen et al., 2016	SE/NO	Quasi-experimental	179 employees in 34 small firms. Gender distribution not specified.	Supportive	Health	Yes (in one subgroup)	No	Not studied
Tafvelin, von Thiele Schwarz & Stenling, 2019	Sweden	Quasi-experimental	37 managers and 538 employees in municipality. 78% women.	Supportive	Work engagement	No	No	Not studied
					Job satisfaction	No	No	Not studied

#### 3.1.1 Full range of leadership model

The Full Range of Leadership Model (FRLM) consists of three leadership styles: transformational, transactional and laissez-faire leadership [39]. According to the theory, transformational and transactional leadership, in turn, comprise different leadership behaviours. Transformational leadership, for example, concerns a focus on vision and inspiration or showing consideration for employee needs, etc. Thirteen studies in the literature review used FRLM as a theoretical starting point. One of the studies used the complete theory [40], but the others only measured transformational leadership. None of the studies explored the various leadership behaviours – only the composite variables. These studies found associations between transformational leadership and employees’ self-perceived well-being [41–46], job satisfaction [41, 47–50] and work engagement [51]. A negative association was also found between passive management by exception and work ability [41]. Nine studies found no statistically significant associations between transformational leadership and health [40, 41, 52, 53], well-being [47, 48, 54], job satisfaction [42, 46, 53] or work ability [40, 52]. No associations were found regarding contingent reward or laissez-faire leadership in relation to health or work ability [40].

Several of the studies also investigated whether other factors mediate the relationship between leadership and well-being – in other words, whether leadership also has indirect significance. These studies found that factors such as innovation climate [54], intervention leadership [52], conflicts between work and private life [47], meaningful work [42, 45, 46], social support [42], cohesion [42], role conflict [42], role clarity [45], opportunities for development [45], self-efficacy [44, 48] influence and involvement [46], and team efficacy [48] mediated the relationship. Studies have also found that the relationship between transformational leadership and well-being was mediated at one point in time but not at another [44, 45]. In other words, these studies indicate that rather than having a direct influence on employee well-being, leadership seems to impact other factors in the work environment, which in turn influence employee well-being. One study also explored reciprocal associations, i.e. whether employee well-being was associated with subsequently performed transformational leadership, and found such an association [45].

Moderation was investigated in one study [41], which found that the relationship between leadership and well-being applied to Danes but not to immigrants.

#### 3.1.2 Ethics and morals-based leadership

Authentic and servant leadership are two leadership theories revolving around the importance of ethics and morals-based leadership [1, 55, 56]. Three studies in the literature review employed authentic leadership theory. Two studies used the theory of servant leadership. They were both longitudinal studies, but the relationship between leadership and well-being was only tested cross-sectionally in one of them. Both authentic leadership and servant leadership consist of subdimensions, but none of the studies used these subdimensions. The studies found that there were associations between authentic leadership and well-being [43] and job satisfaction [50]. One study found no association for the entire group studied but did find an association between authentic leadership and job satisfaction for one subgroup [57]. The studies on servant leadership found associations with work engagement [58, 59] and life satisfaction [58].

Moderation was investigated in one study [57] which found that the relationship applied to the Philippines but not to Norwegians.

#### 3.1.3 Task-oriented and relationship-oriented leadership as well as LMX

Task-oriented and relationship-oriented leadership is a theory that emerged in the 1950 s and focused on two different styles: the degree of focus on the task and the structure for goal attainment or a focus on the people and the group that will complete the task [60, 61]. Every leader can therefore be classified into different combinations of these two behaviour styles.

Leader-member exchange (LMX) is a theory that emerged in the 1970 s in an effort to focus less on the leader’s behaviour and more on the relationship between leaders and followers, and on the exchange that takes place within this relationship [62].

In this literature review, one study used task-oriented and relationship-oriented leadership theory, and one study used LMX. The study on task-oriented and relationship-oriented leadership explored combinations of leadership styles (high–high, high–low, low–high and low–low) and well-being in terms of sense of coherence, and found no statistically significant associations [63]. The study that used LMX theory found an association between LMX and job satisfaction [50]. None of the studies explored any mediating factors, only the direct relationship between leadership and well-being.

#### 3.1.4 Supportive leadership

Eight studies in this literature review focused on supportive behaviours, often based on two broad survey instruments that measure several different psychosocial work environment factors: QPS Nordic [64] and COPSOQ [65]. The leadership that these instruments measure is not an outright leadership theory; rather, these are empirically developed questions with relevance for employee well-being. Among other things, they measure aspects such as fairness, attention and support. One study differentiated between supportive and development-oriented leadership [66]; the other studies used a composite leadership variable. These studies found an association between supportive leadership and job satisfaction [67], meaning at work [68], well-being [69, 70] and quality of life [71]. Three studies did not find statistically significant associations between supportive leadership and mental health [72], vitality [72], self-reported health [66, 73] or well-being [73].

One study was unclear about the association between supportive leadership and work ability [67]. Three studies also examined whether there is an indirect relationship between leadership and well-being. One study found that leadership and well-being were mediated by social capital [70], one found that leadership was mediated by a supportive climate and health-promoting activities [66], and another found that job satisfaction was mediated by interpersonal (for example, social support) and task-related (for example, influence) resources, and that the relationship between leadership and work ability was mediated by job satisfaction, interpersonal resources and task-related resources [67].

Two studies also reported the findings of quasi-experimental leadership interventions. One intervention was based on enhancing leaders’ knowledge of health-promoting leadership [74]. Among other things, the study measured leadership and well-being before and after the intervention, and the results showed that leadership was rated statistically significantly higher after the intervention for the group in Norway but not in Sweden. The results also showed no statistically significant difference in well-being before and after the intervention. There were no statistically significant differences between the intervention group and the control group. The other intervention aimed to increase the need for supportive leadership of managers, and leadership and well-being were measured before and after the intervention [75]. The results showed no statistically significant difference in supportive leadership before and after the intervention, and no differences in job satisfaction or work engagement between the intervention group and the control group.

#### 3.1.5 Intervention leadership

Four of the studies in the literature review investigated a leadership style called intervention leadership, which is not a developed theory of leadership but rather involves how leaders act and provide support during an ongoing intervention. One study [76] developed a scale to measure intervention leadership, which was also used by another study [52]. The third study was based on transformational leadership, but with questions focused on the specific intervention [77], and the fourth study asked about the extent to which the leader encourages the intervention [78].

One study found that leadership had a statistically significant relationship with health and work ability [52], while others found no statistically significant relationships. The studies also examined the indirect relationship, with two studies finding that the association was mediated by other factors, such as participation [78] and meaningful work, role clarity and social support [76]. Two studies found that intervention leadership had no relationship with job satisfaction [77] or work ability [78], either directly or indirectly. One study also explored reciprocal associations, i.e. whether employee job satisfaction or work ability were associated with subsequently performed intervention leadership, and showed that there was no such association [78].

#### 3.1.6 Other leadership behaviours

Four studies in the literature review used leadership perspectives that do not clearly fit under the other headings. One study investigated what the authors called attentive leadership, which involved the general atmosphere, idea development, appreciation and fairness [79]. One study investigated leadership that promotes health and safety, i.e. leadership pertaining to health and safety issues [80]. One study investigated benevolent leadership, which involved how the leader demonstrates care and goodwill towards employees [81]. The fourth study investigated identity leadership, which involved how leaders shape affinity and identity [50]. All studies found a statistically significant association between leadership and self-reported health [79, 80] and job satisfaction [50, 81]. One study investigated whether a lack of being isolated or alone had a moderating effect, and found that leadership was related to self-reported health when employees perceived that they were part of the workplace [80].

#### 3.1.7 Summary of included quantitative studies

In summary, among the quantitative studies included in the literature review, the most commonly used and studied leadership measurement was transformational leadership, followed by supportive leadership. About half of the investigated associations concerning these two types of leadership were statistically significant, and only a few of these were longitudinal. Even though fewer studies investigated authentic leadership, servant leadership or LMX, they all found statistically significant associations. One important difference, however, is that research using transformational and supportive leadership has progressed and introduced different mediators, while none of the studies using authentic leadership, servant leadership or LMX investigated mediators.

The review also found that the most frequently investigated outcome measure is job satisfaction, followed by well-being. Regardless of leadership perspective, job satisfaction is the outcome with which most studies have found associations. While work engagement, quality of life and meaningfulness have been used to a lesser extent, all papers that used these outcome measures found statistically significant associations. If well-being is considered as a multidimensional phenomenon, where the different scales capture different aspects or dimensions of the phenomenon, it is clear that leadership seems to have an “impact” on work-related aspects and, to a lesser extent, on general aspects.

The review found that 15 papers explicitly investigated mediating factors. Of the 22 investigated associations in which mediating factors were included, 19 associations were mediated, one found no mediation and two did not find associations between leadership and outcomes or mediators. One study was also unclear about whether mediating factors were investigated (relative to the specific relationship between leadership and well-being) [52]. The mediators used vary, but were often different kinds of social working conditions (such as a supportive climate), task-related working conditions (such as autonomy), the individual’s attitude and mindset (such as job satisfaction, meaningfulness and self-confidence) or health-promoting activities and initiatives (such as health check-ups). Note that job satisfaction and meaningfulness were considered aspects of well-being by others, i.e. the relationship between leadership and well-being is mediated by aspects of well-being (which applies to five of the studied associations).

Contextual considerations were found to a limited extent in the included studies. In eleven of the studies, hardly any contextual considerations were made, other than a brief description of the sample [43, 46, 58, 59, 63, 68, 69, 71–73, 77, 80]. In some studies, theoretical reasoning was made about the choice of leadership theory and why it suited the sample or population [44, 45, 47, 48, 50, 67, 81]. For example, Nielsen et al. [48] argued that transformational leadership is important to study in the health sector in Denmark because the organization of this sector requires motivational leadership.

In some studies, the Nordic context was highlighted when the authors discussed the generalizations that can be made. Most of these studies described this as a limitation, i.e. that the results need to be replicated and confirmed in other populations and contexts [40, 42, 44, 51, 53, 54, 76], but one study suggested that an advantage of the study was that it was only conducted in one context as it creates a more homogeneous sample [66]. Others highlighted contextual factors as something that needs to be considered more in future studies [47, 52, 74, 75, 78, 79]. For example, Munir et al. [47] mentioned that policies and regulations have not been taken into account in their study, which may be relevant to the examined relationship.

Further, two studies used the Nordic context, such as legislation or culture, to understand and explain their findings. They point out that the association they observed in their studies can be explained by the fact that the legislation obliges managers to work with employees’ job satisfaction [49], and that the individualistic culture of Denmark makes transformational leadership appealing to employees, as it involves providing individual attention [42].

Finally, there were three studies that in their design, sample and variables adjusted for [70] or focused on the context and the influence of context [41, 57]. One study deliberately adjusted for cultural differences to refine the relationship between leadership and employee well-being [62], while two studies showed that ethnicity was a moderator in this relationship [41, 57].

### 3.2 Qualitative studies

The qualitative studies are presented in the form of an overview table (Table 2), a summary of each study and a comparative analysis of patterns in the results.

**Table 2:** Qualitative studies

Paper	Country	Method and sample	Focus
Landstad et al., 2017	SV/NO	18 interviews with 10 Swedish and 8 Norwegian managers at 18 small companies in rural areas.	Study how managers in small companies view health-promoting leadership.
Lundqvist et al., 2012	Sweden	42 interviews with managers at different levels in a manufacturing industry.	Investigate the relationship between managers’ leadership and their health.
Poulsen & Ipsen, 2017	Denmark	Case studies based on 17 interviews with 4 managers and 13 employees in four Danish industries (data/IT, engineering, management, manufacturing).	Investigate how managers ensure employee well-being and organisational performance across geographic distance and in terms of time.
Schön Persson et al., 2018	Sweden	27 interviews with 4 managers and 23 employees in municipal health care practices.	Obtain improved understanding of positive relationships between employees and managers in municipal health care.
Skarholt et al., 2016	Norway	63 interviews with 18 managers and 45 employees as well as meeting observations in four organisations: oil and gas (14 interviews), construction (21 interviews), cleaning (12 interviews), and health care (16 interviews).	Study what leaders do in the workplace to promote health.

#### 3.2.1 Summary of the qualitative studies

Five studies in the literature review used qualitative methodologies to investigate leadership and employee well-being [82–86]. All used interviews to collect data, but Skarholt et al. [86] also conducted observations. Three studies interviewed both managers and employees [84–86], whereas two studies only interviewed managers [82, 83]. Regarding theoretical starting points, the first included study [82] was not based on an explicit leadership theory, but transformational leadership was referenced as being supportive of health. Instead, the study used the concept of “Workplace Health Management”, which was defined in part as a set of leadership behaviours that continually interact with the work environment to shape a setting that improves employee health, and in part as an intentional integration of all company processes to maintain and promote employee health and well-being. The primary focus in the study by Lundqvist et al. [83] was not on what managers do to promote employee well-being; rather, this was an aspect that emerges in the results. The study did not use a leadership theory as a starting point. Nor was there an explicit theory on well-being. Poulsen and Ipsen’s [84] study had no explicit leadership theory guiding the analysis, but in the review of previous research, transformational leadership was raised as a positive form of leadership. The study employed a definition of well-being that included both physical and mental work environments. The study by Schön Persson et al. [85] did not use a specific leadership theory. Concerning health, the authors’ work was based on a salutogenic perspective. The fifth included study [86] was theoretically based on transformational leadership and health-promoting leadership, as well as a salutogenic perspective of health.

Regarding limitations of the studies, three of the studies mentioned the generalisability of the result as a potential limitation, as the data was collected in a specific context [82–84]. In the study by Schön Persson et al. [85], the authors emphasised that the choice not to focus on the significance of relationships of structural and organisational aspects was a weakness. However, they still pointed out that the study results could probably be generalised to contexts other than a Swedish healthcare organisation, as relationships are central regardless of professional category and culture. In the study by Skarholt et al. [86], the authors did not point out any limitations of the study themselves, but they did note that they studied Norwegian workplaces and that leadership there is influenced by Scandinavian leadershippractices.

#### 3.2.2 Overall patterns in the qualitative studies

Regarding patterns in respondents’ views of leadership that promote well-being, four overarching categories can be discerned in the results of the included studies: 1) direct leadership, 2) indirect leadership, 3) mutual influence and 4) leadership adapted to the situation (see Table 3).

**Table 3:** Overarching categories

Category	Description	Examples	Papers
Direct leadership	•Be available and nearby •Create trust and autonomy, delegate •Involve, include •Inspire, motivate	“Hands on”, visit employees regularly, let employees make decisions, include, lead through values to inspire and motivate.	Landstad et al., 2017; Lundqvist et al., 2012; Poulsen & Ipsen, 2017; Schön Persson et al., 2018; Skarholt et al., 2016
Indirect leadership	Create a good and safe physical and psychosocial work environment and facilitate initiatives that can promote health	Loyalty, confidence, trust, and happiness. Wellness activities, variation in work tasks, ergonomic modifications at work.	Landstad et al., 2017; Poulsen & Ipsen, 2017; Skarholt et al., 2016
Mutual influence	Leadership that promotes employee health also has a positive impact on the manager	Promoting health brings satisfaction to managers, and a manager who experiences good health is more interested in employee health.	Landstad et al., 2017; Lundqvist et al., 2012; Schön Persson et al., 2018
Leadership adapted to the situation	Leadership for health and well-being is context- and situation-dependent	Leadership is adapted to contextual factors and the various needs of individuals.	Landstad et al., 2017; Poulsen & Ipsen, 2017; Schön Persson et al., 2018; Skarholt et al., 2016

The first category is about more direct leadership in terms of how the leader or manager behaves in relation to the employees. Relationship-oriented and communicative leadership were common terms for this kind of leadership, but it is also possible to discern four subcategories. First, several authors noted that leadership should be based on availability and proximity, i.e., leaders should be “hands-on” and spend time with employees, instead of working through systems and procedures [83, 84, 86]. Second, leaders need to show trust in employees by delegating tasks and areas of responsibility and by giving them autonomy [82–84]. Third, there is an emphasis on participation, in the sense that leaders involve employees in decision-making and problem-solving processes; that they act democratically and inclusively; and that they validate employees [82, 83, 85, 86]. Fourth, leaders should inspire and motivate employees, for example by leading through values [83, 86].

The second category involves how leaders influence employee health and well-being through indirect leadership. In this area, there are two primary foci. One is to work to achieve a good, safe physical and psychosocial work environment characterised by loyalty, confidence, trust and happiness [82, 86]. The second is to facilitate initiatives that can foster employee health and well-being, such as covering the cost of wellness activities, ensuring variation in work tasks to reduce physical load, implementing ergonomic modifications at work, and collecting data on employee health via surveys [82, 84, 86].

The third category is about mutual influence, which refers to the fact that leadership that promotes employee well-being also has a positive impact on managers. According to Schön Persson et al. [85], managers can foster employee well-being by validating them and involving them in decision-making, which in turn gives managers greater satisfaction and thus improves their own work situation. On the same theme, Lundqvist et al. [83] demonstrated in their study that a manager who experiences well-being was more interested in employee well-being. Furthermore, Landstad et al. [82] concluded that managers must be role models and practise what they preach.

The fourth category is about the apparent lack of any uniform responses to the question of what leadership promoting well-being entails, because it depends largely on the situation and context in which it is performed. The study by Skarholt et al. [86] discussed the fact that the leadership promoting employee well-being did not look the same in the case studies because of the different contextual factors, such as structure, culture and the nature of the work. According to Schön Persson et al. [85], what can be characterised as a health-promoting relationship between managers and employees differed depending on the situation; the manager may need to be outside the group in some cases and more involved and part of the group in others. The study by Poulsen and Ipsen [84] also pointed out that different people need different leadership styles. In other words, it is important to remember that leadership is not a one-way process; as concluded by Landstad et al. [82], it is also important for employees to take personal responsibility for their health and well-being.

Regarding the importance attached to factors that have to do with the more general Nordic context, it can be stated that this was not particularly prominent in any of the studies examined. This is not surprising as none of the studies has expressed such ambitions. However, two of the background descriptions of the studies [82, 86] highlighted certain contextual conditions that can be linked to the Nordic context, such as the existence of legislation that creates conditions for employee participation and a strong tradition of democratization in working life and well-developed collaborations between employers and employees.

The selection of studied organizations in all included qualitative studies does not seem to have been made to specifically examine contextual conditions in the Nordic countries. Rather, the selection seems to have more to do with which countries the researchers are active in. An exception is Landstad et al. [82], who chose to study small companies as these make up a large proportion of the total number of companies in the Nordic region.

Regarding the results, it can be stated that although it was only one study that explicitly linked the identified leadership (democratic) to the Nordic leadership tradition [86], there were empirical patterns in three of the other studies [82, 84, 85] that are in line with the characteristics often mentioned for leadership in the Nordic context, e.g. solidarity, a high degree of personal responsibility among employees, a small power distance between manager and employee and the importance of trust. Furthermore, the study by Landstad et al. [82] also included results that pointed to the importance of work environment legislation which states that managers are responsible for performing systematic occupational health and safety reviews.

## 4 Discussion

This literature review has studied research investigating leadership behaviours that contribute to well-being in the workplace in a Nordic working life context.

To summarize, a direct significance of leadership for employees’ well-being was found in 28 of the quantitative studies. Most studies that investigated the indirect significance of leadership found that this relationship was mediated by other factors. The qualitative studies also emphasised indirect leadership with similar factors to those found in the quantitative studies. These factors concerned the work tasks and conditions for completing them, as well as the social climate and environment in the workplace or the organisation. These factors are consistent with the factors identified in previous literature reviews [14]. Thus, the results of this literature review together with previous literature reviews demonstrate that leadership has significance for well-being among employees, but primarily via other factors in the work environment or the individual. Some connections are made to the Nordic working life context in the reviewed studies, but these connections are not fully elaborated.

The 40 analysed studies used different leadership theories but the Full Range of Leadership Model clearly dominates the field, albeit with only one of the model’s three styles: transformational leadership. It could be the case that certain leadership behaviours are directly related, and others are indirectly related to well-being, but this has not been adequately explored, as it is rare for multiple leadership styles or behaviours to be studied at once. The studies in which different health outcomes were used in relation to mediators provide some insight. In some studies, such as that carried out by Munir et al. [47], transformational leadership was directly related to job satisfaction, while the relationship with well-being was mediated. The association may simply differ depending on what is being measured. Previous literature reviews have also called attention to this [11, 13, 14].

Concerning problems with the theoretical foundation of transformational leadership, and how it has been applied in the included studies, this review can point to several issues. First, it is problematic that the quantitative studies investigated transformational leadership as an overarching style without breaking it down into its four leadership behaviours. Second, it is also problematic that, apart from one paper, the studies did not investigate transformational leadership in relation to the entire theory, the Full Range of Leadership Model (FRLM), which also includes transactional leadership and laissez-faire leadership. This is the case even though the author of the theory considers it a comprehensive theory [39] and other researchers [87] have shown that a combination of transformational and transactional behaviours may be preferable depending on the situation. This theoretical selection may therefore be questionable because the researchers are studying individual styles without a clear theoretical basis for how they are related (also see Arnold [13] for similar criticism). Third, it is important to note that most of the studies included no critique of FRLM. Earlier research has demonstrated methodological shortcomings and the fact that the theory overemphasises the role of the leader and fails to note the significance of employees’ roles as co-creators of leadership has also garnered criticism [16, 17, 88, 89].

Several of the studies used broad theories such as FRLM as their starting points, and consequently, it is very difficult to determine how leadership promotes well-being, and what a manager or leader should actually do, based on the studies’ findings. In other words, it is difficult to transform the results of the quantitative studies into practical action, because the investigated theories are too abstract. The results of the qualitative studies may therefore complement the quantitative studies and clarify the behaviours more precisely. However, the reviewed qualitative studies have other shortcomings, primarily regarding the scope of the results. While the descriptions of leadership are closer to reality in terms of how it is performed, the significance of a few respondents’ experiences of their respective organisations can be questioned. There is also reason to be cautious regarding the qualitative studies that make assertions about the kind of leadership that promotes employee well-being. More specifically, these studies have not investigated the actual outcome in terms of whether employee well-being has indeed been impacted by leadership.

The significance of context for the relationship between leadership and well-being is another problematic aspect. Studying intervening factors, such as mediators, is certainly a step towards contextualising the phenomenon, but collectively, the contextual framework is still underdeveloped in the included studies, especially in the quantitative studies. This problem has also been addressed in earlier literature reviews [9–14]. The lack of context in the studies is problematic because the unique aspects of the study material are neither analysed nor problematised, and knowledge of how organisational factors (such as work environment policies) or national factors (such as the Swedish model, the Co-Determination in the Workplace Act [MBL], or work environment provisions [19–21]) shape the relationship is rendered invisible. Of the quantitative studies in this review, only two [42, 49] related their findings to the Nordic context. There is therefore a risk that too much focus will be placed on individual leaders in the form of their leadership when, in actuality, the focus should be on the organisation. Furthermore, most leadership theories used in the studies were developed in a North American context but were applied relatively uncritically in a Nordic context. The risk of theoretical reproduction thus becomes imminent, i.e. North American theories are confirmed in a Nordic context because the unique aspects of the Nordic context are not factored in. An example of this is that few studies discussed the significance of the population from which the data had been collected. As demonstrated by the results of the review, the material in many of the studies was from the social services sector and the respondents were predominantly female. In other words, there is a potential risk of bias.

Contextual aspects may be more easily captured in the qualitative studies, especially when they are analysed inductively, as they do not have to start from or limit themselves to these typically North American-influenced theories. In the reviewed studies, however, the problem is rather reversed, as several behaviours were identified but without any developed theory or explicit connections to the Nordic context. The qualitative studies did, however, show several common patterns. The behaviours of leaders and managers identified in the qualitative studies as promoting employees’ well-being largely recur in the leadership theories and scales for leadership used in the quantitative studies, such as transformational leadership [26, 39]. However, the qualitative studies identified the need for leadership that is adapted to the prevalent situation, a theme that was rarely found in the quantitative studies. These findings suggest that leaders are expected to be available and to provide active help and support to employees, while also being sufficiently distanced to provide space and a mandate, and not to interfere. This could be a case where the Nordic context shines through, in terms of Scandinavian leadership as noted in one of the studies [86]. Here, once again, it is problematic that the quantitative studies do not go into sufficient depth, for instance, by considering situational moderators or applying a design with frequent measuring. It could be the case that some leadership behaviours are important in certain situations, while others are more important in different ones.

Although previous literature reviews [8–14] have pointed out similar shortcomings in research about the importance of leadership for employee well-being, this review makes several new contributions. First, a contribution is made by including both quantitative and qualitative studies, where the previous reviews only included quantitative, as has been requested previously [10]. In this way, more perspectives are incorporated and provide a broader picture of the phenomenon. Second, this review focuses on contextual considerations made in the studies in the research field, something that has not received thorough examination before. Previous literature reviews [8, 10–14] have highlighted the need for further studying moderators of the relationship between leadership and well-being, but our review shows that the lack of contextual considerations in previous studies also clarifies the theoretical problems that exist when applying theories to different cultural contexts. Third, a contribution with this review compared to previous reviews [8–14] is thus the critique that is directed not only at methods used in previous research in the field but also the choice of and practical application of leadership theory.

### 4.1 Suggested areas for future research

Several issues have been identified in the existing research. Beginning with methodology, it can be concluded that the dominance of quantitative studies has resulted in a great deal of information about the occurrence of leadership fostering well-being but relatively little knowledge of what this entails in the day-to-day work. Put simply, we know that transformational leadership is beneficial to employee well-being, but we do not know how a manager performs this kind of leadership in practice. Thus, learning more about the leadership practised requires a different kind of data collection, such as observing managers and employees in daily work. Research on what managers do includes several well-conducted studies based on shadowing in the field, meeting observations and contextual interviews [90, 91]. Such methods could facilitate a better understanding of actual leadership practices. Furthermore, case studies would be suitable for counteracting the lack of contextualisation that characterises many of the reviewed studies. One advantage of case studies is that it is natural to capture leadership in context, i.e., to create rich descriptions of how the surrounding factors influence managers’ and leaders’ opportunities to exercise their leadership. The information that could be generated through case studies could then be verified through quantitative-oriented studies.

Another issue related to method is the lack of knowledge regarding the long-term influence of leadership on employees’ well-being. Thus, there is a need for longitudinal, multi-method studies to investigate the ways in which leadership influences employee well-being and whether this changes over time. This is not a novel finding of this literature review; similar inadequacies have been identified and possible actions presented in all previous literature reviews of this subject [8–14].

The last methodological issue concerns the often-homogeneous material collected in the different papers. There is a need for broader, comparative studies, where several types of industries and organisational sizes are represented in order to identify common patterns and contextual differences.

Regarding theoretical issues, a small number of theories have been granted enormous significance in the field. These are, however, often not problematised and the field is rather focused on the leader. Theories about co-workership and co-leadership, for example, could contribute a new understanding of how leadership is generated and maintained. Based on theories of gender, diversity and equality, we could probably also discover several aspects of the relationship between leader and employee that could contribute to a more nuanced picture of leadership promoting well-being.

### 4.2 Practical implications

Based on the reviewed articles and previous research, several potential implications can be identified.

Despite the challenges addressed previously, the reviewed research does have some level of consensus regarding overarching leadership behaviours that may work well for fostering well-being. For example, these behaviours include being a role model for employees with regard to work and health, and also inspiring and motivating them at work. It is also important to encourage employees’ personal development. Furthermore, it is important to be available, to show trust and to give employees space and autonomy. As it may be difficult to find a balance between being present and supportive, while also providing space and responsibility, it is important for leaders and employees to have a continual dialogue about their expectations of leadership, so that the leader can adapt to the needs of employees and the organisation. An employee who prefers to be given space for one task may need a more present leader for another task. This requires flexible leadership that is adapted to the current situation and context.

It is also important to point out that it has proven difficult to capture exactly how the leadership behaviours described above actually impact employee well-being. Rather, the research often points to the significance of indirect leadership, for example by building a culture and an environment that foster health. What this culture or environment looks like depends on several factors such as leeway, resources, the task, expertise and so forth. This necessitates discussions regarding what well-being means in the workplace and what the expectations are of leaders and colleagues in this regard.

### 4.3 Methodological challenges and limitations of the literature review

To increase the transparency of this literature review, and to present to the reader with the most objective picture possible, we want to comment on a few challenges posed by this kind of review and the process of comparing studies. Concepts such as well-being and leadership are theoretically complex and difficult to operationalise. This has entailed different studies approaching the subject from different perspectives and using several different terms and instruments. We have tried to clarify what the authors studied, but the terms may have been defined differently or measured with different instruments. In other words, there is a risk that they are capturing or measuring different aspects of the phenomenon in question.

Furthermore, although the quantitative studies focused on the relationship between leadership and well-being, their final models may have contained different variables. Some adjusted for different background factors and several other work environment factors, while others adjusted for a few factors, such as gender and age. Therefore, both bivariate associations (without adjusting for other factors) and adjusted associations are reported to give the reader a clearer and fairer picture.

The studies in the literature review also differ regarding the number of participants included in the analysis. For example, one study is based on material from over 29,000 participants, while another study had just over 100 participants. This is significant for the studies’ likelihood of detecting statistically significant associations. With many participants, it is easier to find a statistically significant association, even if the association is weak. Some studies report associations that are very low but still statistically significant, probably because there are several thousand participants. However, in most studies, the association was about 0.15 to 0.25, which means leadership explains about 2 to 6 percent of employee well-being.

The review presents two studies as intervention studies, but in reality, several studies were based on material from interventions. However, these studies lacked a clear control group and focused more on the significance of leadership for the outcome of well-being, among other things, and less on the evaluated intervention. They were therefore presented as association studies.

With regard to the qualitative studies, it is striking that only one of the studies asked employees about how they experience their well-being [84]. In other words, it is impossible for the authors of the other studies to express whether the identified leadership behaviours actually impact the well-being of employees. Moreover, none of the studies were based on a specific leadership theory, and it is therefore hard to judge the theoretical contribution of the study findings.

When it comes to the potential scope of qualitative studies, it is important to remember that the aim here is not statistical generalisation as with the quantitative studies, but so-called analytical generalisation, i.e., expanding and generalising theories [92] or generalising via context similarity [93]. Thus, the qualitative studies in this literature review should not only be understood as a complement to the quantitative studies but can also stand independently.

## 5 Conclusions

The studies included in this literature review suggest that leadership is related to employee well-being, although this relationship seems to be indirect, mediated by other factors in the working environment. Transformational leadership and supportive leadership seem to have associations with employee well-being, especially in relation to work-related outcomes, such as job satisfaction and work engagement. Further, relationship-oriented and democratic leadership, characterised by a leader who motivates and inspires employees, who is available and listens to employees, and who simultaneously shows trust in employees’ abilities by giving them responsibility, space and codetermination may also promote employee well-being.

The fact that the research was carried out in the Nordic countries was only considered to a limited extent, both in terms of included variables and in the discussion of the research results. This lack of consideration for the Nordic context is problematic as potentially unique aspects of the study material is rendered invisible. Leadership that is adapted to the prevalent situation in terms of leaders being available while simultaneously showing trust in employees and giving them space and a mandate is a theme found primarily in the qualitative studies. Thus, quantitative and qualitative methods together provide a clearer picture of the kinds of leadership behaviours that promote well-being, especially in the Nordic working life context.

The literature review also demonstrates a need for more research in the field. To obtain a better understanding of the relationship between leadership and well-being and how such leadership should be exercised in practice, the significance of context must be studied more, and different kinds of specific leadership behaviours must be compared. More longitudinal studies that use and combine material from different sources and apply different theoretical perspectives are needed.

## Ethical approval

Not applicable.

## Informed consent

Not applicable.

## Conflict of interest

The authors declare no conflict of interest.

## References

[1] Northouse PG . Leadership: theory and practice. Seventh Edition. Los Angeles: SAGE Publications, Inc; 2015. 494 p.

[2] Yukl G . Leadership in organizations. 8th ed. Boston: Pearson; 2013. 511 p.

[3] Nielsen K , Taris TW . Leading well: Challenges to researching leadership in occupational health psychology–and some ways forward. Work Stress. 2019;33(2):107–18.

[4] Eriksson A . Health-Promoting Leadership: [Dissertation]. Nordic School of Public Health; 2011.

[5] Karasek R , Theorell T . Healthy work: stress, productivity, and the reconstruction of working life. New York, NY: Basic Books; 1990. 381 p.

[6] Nyberg A . Det goda chefskapet. Organisatorisk effektivitet och anställdas hälsa. En kunskapsöversikt. In: Chefskapets former och resultat Stockholm: VINNOVA; 2008.

[7] Nyberg A . The impact of managerial leadership on stress and health among employees [Disseration]. Karolinska Institutet; 2009.

[8] Nyberg A , Bernin P , Theorell T . The impact of leadership on the health of subordinates. Stockholm, Sweden: National Institute for Working Life; 2005. (SALTSA). Report No.: 1:2005.

[9] Kuoppala J , Lamminpää A , Liira J , Vainio H . Leadership, Job Well-Being, and Health Effects—A Systematic Review and a Meta-Analysis: J Occup Environ Med. 2008;50(8):904–15. 18695449 10.1097/JOM.0b013e31817e918d

[10] Skakon J , Nielsen K , Borg V , Guzman J . Are leaders’ well-being, behaviours and style associated with the affective well-being of their employees? A systematic review of three decades of research. Work Stress. 2010;24(2):107–39.

[11] Harms PD , Credé M , Tynan M , Leon M , Jeung W . Leadership and stress: A meta-analytic review. Leadersh Q. 2017;28(1):178–94.

[12] Montano D , Reeske A , Franke F , Hüffmeier J . Leadership, followers’ mental health and job performance in organizations: A comprehensive meta-analysis from an occupational health perspective. J Organ Behav. 2017;38(3):327–50.

[13] Arnold KA . Transformational leadership and employee psychological well-being: A review and directions for future research. J Occup Health Psychol. 2017;22(3):381–93. 28150998 10.1037/ocp0000062

[14] Inceoglu I , Thomas G , Chu C , Plans D , Gerbasi A . Leadership behavior and employee well-being: An integrated review and a future research agenda. Leadersh Q. 2018;29(1):179–202.

[15] Dinh JE , Lord RG , Gardner WL , Meuser JD , Liden RC , Hu J . Leadership theory and research in the new millennium: Current theoretical trends and changing perspectives. Leadersh Q. 2014;25(1):36–62.

[16] Yukl G . An evaluation of conceptual weaknesses in transformational and charismatic leadership theories. Leadersh Q. 1999;10(2):285–305.

[17] Tourish D , Pinnington A . Transformational Leadership, Corporate Cultism and the Spirituality Paradigm: An Unholy Trinity in the Workplace? Hum Relat. 2002;55(2):147–72.

[18] Alvesson M . Upbeat leadership: A recipe for – or against – “successful” leadership studies. Leadersh Q. 2020;31(6):101439.

[19] Asheim BT . Learning, Innovation and Participation: Nordic Experiences in a Global Context with a Focus on Innovation Systems and Work Organization. In: Ekman M, Gustavsen B, Asheim BT, Pålshaugen Ø, editors. Learning Regional Innovation. London: Palgrave Macmillan UK; 2011. p. 15–49.

[20] Bengtsson M , Berglund T . Negotiating alone or through the union? Swedish employees’ attitudes in 1997 and 2006. Econ Ind Democr. 2011;32(2):223–41.

[21] Visser J . The quality of industrial relations and the Lisbon strategy. In: Visser J, editor. Industrial relations in Europe 2008. Luxembourg: Office for Official Publications of the European Communities; 2009.

[22] Holmberg I , Åkerblom S . Modelling leadership—Implicit leadership theories in Sweden. Scand J Manag. 2006;22(4):307–29.

[23] Andreasson U , Lundqvist M , Ministerråd Nordisk . Nordic leadership. Nordic Council of Ministers; 2019.

[24] Grenness T . Scandinavian Managers on Scandinavian Management. Int J Value-Based Manag. 2003;16(1):9–21.

[25] Larsson G , Carlstedt L , Andersson J , Andersson L , Danielsson E , Johansson A , et al. A comprehensive system for leader evaluation and development. Leadersh Organ Dev J. 2003;24(1):16–25.

[26] Bass BM . Leadership and performance beyond expectations. New York: Free Press; 1985. 256 p.

[27] Larsson G , Hyllengren P . Contextual influences on leadership in emergency type organisations: Theoretical modelling and empirical tests. Int J Organ Anal. 2013;21(1):19–37.

[28] Nielson P . Arbejdsliv i Norden. Nordic Council of Ministers; 2016.

[29] Christensen JO , Bakke Finne L , Kristiansen J . The future of the Nordic psychosocial work environment: Implications for occupational health. Nordic Council of Ministers; 2021.

[30] Alsos K , Dølvik JE . The Future of Work in the Nordic countries: Opportunities and Challenges for the Nordic Life Models. Nordic Council of Ministers; 2021.

[31] Grant MJ , Booth A . A typology of reviews: an analysis of 14 review types and associated methodologies: A typology of reviews. Health Inf Libr J. 2009;26(2):91–108. 10.1111/j.1471-1842.2009.00848.x 19490148

[32] Torraco RJ . Writing Integrative Literature Reviews: Guidelines and Examples. Hum Resour Dev Rev. 2005;4(3):356–67.

[33] Moher D . Preferred Reporting Items for Systematic Reviews and Meta-Analyses: The PRISMA Statement. Ann Intern Med. 2009;151(4):264. 19622511 10.7326/0003-4819-151-4-200908180-00135

[34] Page MJ , McKenzie JE , Bossuyt PM , Boutron I , Hoffmann TC , Mulrow CD , et al. The PRISMA 2020 statement: an updated guideline for reporting systematic reviews. BMJ. 2021;n71. 33782057 10.1136/bmj.n71 PMC8005924

[35] Tompa E , Trevithick S , McLeod C . Systematic review of the prevention incentives of insurance and regulatory mechanisms for occupational health and safety. Scand J Work Environ Health. 2007;33(2):85–95. 17460796 10.5271/sjweh.1111

[36] Tompa E , Kalcevich C , Foley M , McLeod C , Hogg-Johnson S , Cullen K , et al. A systematic literature review of the effectiveness of occupational health and safety regulatory enforcement: Review of OHS Regulatory Enforcement. Am J Ind Med. 2016;59(11):919–33. 27273383 10.1002/ajim.22605

[37] SBU. SBUs handbok - Utvärdering av metoder i hälso- och sjukvården och insatser i socialtjänsten. Statens beredning för medicinsk och social utvärdering; 2017.

[38] Hsieh H-F , Shannon SE . Three Approaches to Qualitative Content Analysis. Qual Health Res. 2005;15(9):1277–88. 16204405 10.1177/1049732305276687

[39] Bass BM , Riggio RE . Transformational leadership. 2nd ed. Mahwah, N.J: L. Erlbaum Associates; 2006. 282 p.

[40] Hasson H , von Thiele Schwarz U , Tafvelin S . Shared or different realities: Self–other agreement on constructive and passive leadership and employee outcomes. Leadersh Organ Dev J. 2019;41(1):37–51.

[41] Holten AL , Bollingtoft A , Carneiro IG , Borg V . A within-country study of leadership perceptions and outcomes across native and immigrant employees: Questioning the universality of transformational leadership. J Manag Organ. 2018;24(1):145–62.

[42] Nielsen K , Daniels K . Does shared and differentiated transformational leadership predict followers’ working conditions and well-being? Leadersh Q. 2012;23(3):383–97.

[43] Perko K , Kinnunen U , Tolvanen A , Feldt T . Investigating occupational well-being and leadership from a person-centred longitudinal approach: congruence of well-being and perceived leadership. Eur J Work Organ Psychol. 2016;25(1):105–19.

[44] Nielsen K , Munir F . How do transformational leaders influence followers’ affective well-being? Exploring the mediating role of self-efficacy. Work Stress. 2009;23(4):313–29.

[45] Nielsen K , Randall R , Yarker J , Brenner S-O . The effects of transformational leadership on followers’ perceived work characteristics and psychological well-being: A longitudinal study. Work Stress. 2008;22(1):16–32.

[46] Nielsen K , Yarker J , Brenner S-O , Randall R , Borg V . The importance of transformational leadership style for the well-being of employees working with older people. J Adv Nurs. 2008;63(5):465–75. 18727749 10.1111/j.1365-2648.2008.04701.x

[47] Munir F , Nielsen K , Garde AH , Albertsen K , Carneiro IG . Mediating the effects of work-life conflict between transformational leadership and health-care workers’ job satisfaction and psychological wellbeing. J Nurs Manag. 2012;20(4):512–21. 22591153 10.1111/j.1365-2834.2011.01308.x

[48] Nielsen K , Yarker J , Randall R , Munir F . The mediating effects of team and self-efficacy on the relationship between transformational leadership, and job satisfaction and psychological well-being in healthcare professionals: A cross-sectional questionnaire survey. Int J Nurs Stud. 2009;46(9):1236–44. 19345946 10.1016/j.ijnurstu.2009.03.001

[49] Tafvelin S , Hasson H , Holmström S , von Thiele Schwarz U . Are Formal Leaders the Only Ones Benefitting From Leadership Training? A Shared Leadership Perspective. J Leadersh Organ Stud. 2019;26(1):32–43.

[50] van Dick R , Lemoine JE , Steffens NK , Kerschreiter R , Akfirat SA , Avanzi L , et al. Identity leadership going global: Validation of the Identity Leadership Inventory across 20 countries. J Occup Organ Psychol. 2018;91(4):697–728.

[51] Mauno S , Ruokolainen M , Kinnunen U , De Bloom J . Emotional labour and work engagement among nurses: Examining perceived compassion, leadership and work ethic as stress buffers. J Adv Nurs. 2016;72(5):1169–81. 26841277 10.1111/jan.12906

[52] Lundmark R , Hasson H , von Thiele Schwarz U , Hasson D , Tafvelin S . Leading for change: line managers’ influence on the outcomes of an occupational health intervention. Work Stress. 2017;31(3):276–96.

[53] Roczniewska M , Richter A , Hasson H , Schwarz U von T . Predicting Sustainable Employability in Swedish Healthcare: The Complexity of Social Job Resources. Int J Environ Res Public Health. 2020;17(4):1200. 32069935 10.3390/ijerph17041200 PMC7068286

[54] Tafvelin S , Armelius K , Westerberg K . Toward Understanding the Direct and Indirect Effects of Transformational Leadership on Well-Being: A Longitudinal Study. J Leadersh Organ Stud. 2011;18(4):480–92.

[55] Gardner WL , Cogliser CC , Davis KM , Dickens MP . Authentic leadership: A review of the literature and research agenda. Leadersh Q. 2011;22(6):1120–45.

[56] Spears LC , editor. Reflections on leadership: how Robert K. Greenleaf’s theory of Servant-leadership influenced today’s top management thinkers. New York: J. Wiley; 1995. 352 p.

[57] Nielsen MB , Tvedt SD , Matthiesen SB . Prevalence and occupational predictors of psychological distress in the offshore petroleum industry: A prospective study. Int Arch Occup Environ Health. 2013;86(8):875–85. 23099441 10.1007/s00420-012-0825-x

[58] Upadyaya K , Vartiainen M , Salmela-Aro K . From job demands and resources to work engagement, burnout, life satisfaction, depressive symptoms, and occupational health. Burn Res. 2016;3(4):101–8.

[59] Upadyaya K , Salmela-Aro K . Social demands and resources predict job burnout and engagement profiles among Finnish employees. Anxiety Stress Coping. 2020;33(4):403–15. 32223447 10.1080/10615806.2020.1746285

[60] Blake RR , Mouton JS . The Managerial Grid. Houston, TX: Gulf Publ. Co; 1964. 340 p.

[61] House RJ , Aditya R . The Social Scientific Study of Leadership: Quo Vadis? J Manag. 1997;23(3):409–73.

[62] Graen GB , Uhl-Bien M . Relationship-based approach to leadership: Development of leader-member exchange (LMX) theory of leadership over 25 years: Applying a multi-level multi-domain perspective. Leadersh Q. 1995;6(2):219–47.

[63] Svensson S , Stubbs J , Larsson J . The association between subordinate perception of task and relation oriented leadership behaviors and sense of coherence among a sample of Swedish white-collar workers. Work. 2018;61(2):327–36. 30373982 10.3233/WOR-182803

[64] Lindström K , Nordic Council of Ministers. User’s guide for the QPSNordic: general Nordic questionnaire for psychological and social factors at work. Copenhagen: Nordic Council of Ministers; 2000.

[65] Berthelsen H . COPSOQ II - en uppdatering och språklig validering av den svenska versionen av en enkät för kartläggning av den psykosociala arbetsmiljön på arbetsplatser. Stockholm: Stressforskningsinstitutet; 2014.

[66] Ljungblad C , Granström F , Dellve L , Åkerlind I . Workplace health promotion and working conditions as determinants of employee health. Int J Workplace Health Manag. 2014;7(2):89–104.

[67] Berthelsen H , Hakanen JJ , Westerlund H . Copenhagen psychosocial questionnaire - A validation study using the job demand-resources model. PLoS ONE. 2018;13(4). 10.1371/journal.pone.0196450 PMC5927437 29708998

[68] Clausen T , Borg V . Job demands, job resources and meaning at work. J Manag Psychol. 2011;26(8):665–81.

[69] Finne LB , Christensen JO , Knardahl S . Psychological and social work factors as predictors of mental distress and positive affect: A prospective, Multilevel study. PLoS ONE. 2016;11(3). 10.1371/journal.pone.0152220 PMC4807036 27010369

[70] Kizuki M , Fujiwara T . Quality of supervisor behaviour, workplace social capital and psychological well-being. Occup Med. 2020;70(4):243–50. 10.1093/occmed/kqaa070 32421808

[71] Lohela M , Björklund C , Vingård E , Hagberg J , Jensen I . Does a change in psychosocial work factors lead to a change in employee health? J Occup Environ Med. 2009;51(2):195–203. 19209041 10.1097/JOM.0b013e318192bd2c

[72] Burr H , Albertsen K , Rugulies R , Hannerz H . Do dimensions from the Copenhagen Psychosocial Questionnaire predict vitality and mental health over and above the job strain and effort-reward imbalance models? Scand J Public Health. 2010;38(SUPPL. 3):59–68. 21172772 10.1177/1403494809353436

[73] Hagqvist E , Vinberg S , Landstad BJ , Nordenmark M . Is the gap between experienced working conditions and the perceived importance of these conditions related to subjective health? Int J Workplace Health Manag. 2018;11(1):2–15.

[74] Hansen E , Landstad BJ , Gundersen KT , Vinberg S . Leader-Based Workplace Health Interventions - A Before-After Study in Norwegian and Swedish Small-Scale Enterprises. Int J Disabil Manag. 2016;11.

[75] Tafvelin S , von Thiele Schwarz U , Stenling A . Leadership Training to Increase Need Satisfaction at Work: A Quasi-Experimental Mixed Method Study. Front Psychol. 2019;10:2175. 31607994 10.3389/fpsyg.2019.02175 PMC6773884

[76] Nielsen K , Randall R . Managers’ Active Support when Implementing Teams: The Impact on Employee Well-Being. Appl Psychol Health Well-Being. 2009;1(3):374–90.

[77] Lundmark R , von Thiele Schwarz U , Hasson H , Stenling A , Tafvelin S . Making it fit: Associations of line managers’ behaviours with the outcomes of an organizational-level intervention. Stress Health. 2018;34(1):163–74. 28681480 10.1002/smi.2770

[78] Tafvelin S , von Thiele Schwarz U , Nielsen K , Hasson H . Employees’ and line managers’ active involvement in participatory organizational interventions: Examining direct, reversed, and reciprocal effects on well-being. Stress Health. 2019;35(1):69–80. 30303299 10.1002/smi.2841

[79] Westerlund H , Nyberg A , Bernin P , Hyde M , Oxenstierna G , Jäppinen P , et al. Managerial leadership is associated with employee stress, health, and sickness absence independently of the demand-control-support model. Work. 2010;37(1):71–9. 20858989 10.3233/WOR-2010-1058

[80] Nielsen K , Daniels K , Nayani R , Donaldson-Feilder E , Lewis R . Out of mind, out of sight? Leading distributed workers to ensure health and safety. Work Stress. 2019;33(2):173–91.

[81] Nie D , Lämsä AM . Chinese immigrants’ occupational well-being in Finland: the role of paternalistic leadership. Leadersh Organ Dev J. 2018;39(3):340–52.

[82] Landstad BJ , Hedlund M , Vinberg S . How managers of small-scale enterprises can create a health promoting corporate culture. Int J Workplace Health Manag. 2017;10(3):228–48.

[83] Lundqvist D , Fogelberg Eriksson A , Ekberg K . Exploring the relationship between managers’ leadership and their health. Work. 2012;42(3):419–27. 22523020 10.3233/WOR-2012-1395

[84] Poulsen S , Ipsen C . In times of change: How distance managers can ensure employees’ wellbeing and organizational performance. Saf Sci. 2017;100:37–45.

[85] Schön Persson S , Nilsson Lindström P , Pettersson P , Andersson I , Blomqvist K . Relationships between healthcare employees and managers as a resource for well-being at work. Soc Health Vulnerability. 2018;9(1):1547035.

[86] Skarholt K , Blix EH , Sandsund M , Andersen TK . Health promoting leadership practices in four Norwegian industries. Health Promot Int. 2016;31(4):936–45. 26294755 10.1093/heapro/dav077

[87] Vera D , Crossan M . Strategic Leadership and Organizational Learning. Acad Manage Rev. 2004;29(2):222–40.

[88] Alvesson M . Waiting for Godot: Eight major problems in the odd field of leadership studies. Leadership. 2019;15(1):27–43.

[89] Kurtessis JN , Eisenberger R , Ford MT , Buffardi LC , Stewart KA , Adis CS . Perceived Organizational Support: A Meta-Analytic Evaluation of Organizational Support Theory. J Manag. 2017;43(6):1854–84.

[90] Mintzberg H . Managing. Harlow: Financial Times Prentice Hall; 2009.

[91] Tengblad S , editor. The work of managers: towards a practice theory of management. Oxford; New York: Oxford University Press; 2012. 365 p.

[92] Yin RK . Case study research: design and methods. Fifth edition. Los Angeles: SAGE; 2014. 282 p.

[93] Larsson S . A pluralist view of generalization in qualitative research. Int J Res Method Educ. 2009;32(1):25–38.

